# Cold water immersion after a soccer match: Does the placebo effect occur?

**DOI:** 10.3389/fphys.2023.1062398

**Published:** 2023-02-21

**Authors:** Nidhal Nasser, Houssem Zorgati, Hamdi Chtourou, Alexandre Guimard

**Affiliations:** ^1^ Activité Physique, Sport et Santé, UR18JS01, Observatoire National du Sport, Tunis, Tunisie; ^2^ Institut Supérieur du Sport et de l’Education Physique de Sfax, Université de Sfax, Sfax, Tunisie; ^3^ Institut Supérieur du Sport et de l’Education Physique de Gafsa, Université de Gafsa, Gafsa, Tunisie; ^4^ Université Sorbonne Paris Nord, Hypoxie et Poumon, H&P, INSERM, UMR 1272, Bobigny, France; ^5^ Département STAPS, Université Sorbonne Paris Nord, Bobigny, France

**Keywords:** recovery, performance, muscle damage, DOMS, LIST, cryotherapy, football (soccer)

## Abstract

Although cold water immersion (CWI) is one of the most widely used post-exercise strategies to accelerate recovery processes, the benefits of CWI may be associated with placebo effects. This study aimed to compare the effects of CWI and placebo interventions on time course of recovery after the Loughborough Intermittent Shuttle Test (LIST). In a randomized, counterbalanced, crossover study, twelve semi-professional soccer players (age 21.1 ± 2.2 years, body mass 72.4 ± 5.9 kg, height 174.9 ± 4.6 cm, $\dot{V}$ O_2max_ 56.1 ± 2.3 mL/min/kg) completed the LIST followed by CWI (15 min at 11°C), placebo (recovery Pla beverage), and passive recovery (Rest) over three different weeks. Creatine kinase (CK), C-reactive protein (CRP), uric acid (UA), delayed onset muscle soreness (DOMS), squat jump (SJ), countermovement jump (CMJ), 10-m sprint (10 mS), 20-m sprint (20 mS) and repeated sprint ability (RSA) were assessed at baseline and 24 and 48 h after the LIST. Compared to baseline, CK concentration was higher at 24 h in all conditions (*p* < 0.01), while CRP was higher at 24 h only in CWI and Rest conditions (*p* < 0.01). UA was higher for Rest condition at 24 and 48 h compared to Pla and CWI conditions (*p* < 0.001). DOMS score was higher for Rest condition at 24 h compared to CWI and Pla conditions (*p* = 0.001), and only to Pla condition at 48 h (*p* = 0.017). SJ and CMJ performances decreased significantly after the LIST in Rest condition (24 h: −7.24%, *p* = 0.001 and −5.45%, *p* = 0.003 respectively; 48 h: −9.19%, *p* < 0.001 and −5.70% *p* = 0.002 respectively) but not in CWI and Pla conditions. 10 mS and RSA performance were lower for Pla at 24 h compared to CWI and Rest conditions (*p* < 0.05), while no significant change was observed for 20 mS time. These data suggests that CWI and Pla intervention were more effective than the Rest conditions in recovery kinetics of muscle damage markers and physical performance. Furthermore, the effectiveness of CWI would be explained, at least in part, by the placebo effect.

## 1 Introduction

Soccer is a high-intensity interval sport that requires high physical capabilities during match play (Stølen et al., 2005). Indeed, players have to repeat a wide range of physically demanding actions such as sprints, acceleration/deceleration, changes of direction and duels throughout the match. This combination of physical load induces muscle damage due to their eccentric nature and results in impaired muscle metabolism (Rampinini et al., 2011). Therefore, players are likely to experience fatigue over the following hours and days after a single match (Nédélec et al., 2012) or also after a Loughborough Intermittent Shuttle Test (LIST), a protocol designed to simulate the activities of real soccer match play (Thompson et al., 1999). Over the years, the number of training sessions and competitions per season has steadily increased to become very high up to 60–80 games for some players in the 2017–2018 season which ended with the International Association Football Federation (FIFA) World Cup in Russia. As a result, periods of match congestion (*i.e.*, 1–3 matches per week) are common in elite soccer (Anderson et al., 2016). These activities limit recovery time and can lead to accumulated fatigue and consequently reduced performance capacity (Nédélec et al., 2012). Hence there is a need to use strategies to accelerate the recovery process and minimize the effects of the previous session (competition and/or training), thus reducing the risk of muscle injury (Nédélec et al., 2012; Leeder et al., 2014).

Several strategies are proposed to accelerate recovery from exercise-induced muscle damage (EIMD). All of these strategies are documented in the literature and currently implemented in professional soccer clubs to improve recovery (Nédélec et al., 2012). Indeed, soccer players often use compression garments (Hill et al., 2014), massage (Best et al., 2008), antioxidant supplementation (Howatson et al., 2010), and cryotherapy (Hohenauer et al., 2015). One form of cryotherapy is cold water immersion (CWI). CWI has become the most popular recovery strategy in soccer due to its ease of implementation and low cost. Despite the popularity of CWI and its increased use in soccer, the results of its benefits remain unclear and the data regarding its effectiveness are ambiguous (Ihsan et al., 2016; Broatch et al., 2018; Moore et al., 2022). Indeed, some studies have demonstrated a beneficial effect of CWI on the recovery of physiological and physical parameters (Wilcock et al., 2006; Ascensão et al., 2011; Elias et al., 2012; Bouzid et al., 2018). While other studies report little or no effect of CWI on the recovery of these parameters (Rupp et al., 2012; Leeder et al., 2015; Leeder et al., 2019). These conflicting findings can be attributed to the different methodologies used (Vieira et al., 2016). Indeed, there is a multitude of possible combinations between temperature, time and depth in the immersion protocol. Using LIST to induce muscle damage, Bouzid et al. (2018) found accelerated recovery on different physical performance [countermovement jump (CMJ), squat jump (SJ), 20 m sprint (20 mS)] and muscle damage markers [creatine kinase (CK)] with CWI (10 min at 10°C) compared to thermoneutral water immersion (TWI) (10 min at 28°C) in professional soccer players. In addition, CWI (10 min at 10°C) enhanced recovery of muscle soreness, maximal voluntary contraction (MVC) and myoglobin concentration compared to passive recovery after LIST (Bailey et al., 2007). Similarly, CWI (10 min at 10°C) had a positive effect on recovery of 20 mS and plasma lactate dehydrogenase (LDH) compared with TWI (10 min at 28 ± 2°C) after LIST (Bouchiba et al., 2022). In contrast, Corbett et al. (2012) reported no significant effect on recovery of muscle soreness, MVC, CK activity and myoglobin concentrations in CWI (12 min at 12°C) compared with TWI (12 min at 35°C) and active recovery (12 min walking at 5 km h^-1^) after LIST. Moreover, sitting and standing CWI (14 min at 14°C) did not alter physical performance recovery kinetics (CMJ and MVC), CK and C-reactive protein (CRP) compared to passive recovery after LIST (Leeder et al., 2015).

To explain the accelerated recovery after CWI, several physiological mechanisms have been proposed. Indeed, the improved post-exercise recovery by CWI has been attributed to vasoconstriction, resulting from the exposure of the muscle to cold, which can limit the permeability of vessels and therefore, inflammatory processes, thus reducing muscle pain and edema formation from muscle damage (Bailey et al., 2007; Peake et al., 2017). In addition to the effect of cold, hydrostatic pressure acts on the body and its physiological changes include intracellular-intravascular fluid shifts, reduction of muscle edema and perception of muscle soreness (Wilcock et al., 2006). Furthermore, Ihsan et al. (2016) found that hydrostatic pressure combined with a reduction in muscle temperature by CWI induced changes in blood flow. Some authors suggested that the reduction in blood flow may reduce metabolite accumulation, muscle damage and muscle soreness (Bleakley and Davison, 2010; Leeder et al., 2012). Moreover, the involvement of psychological mechanisms in the effectiveness of CWI should not be overlooked. Indeed, given the popularity of CWI as a good recovery strategy can be somewhat detached from water pressure or temperature, there could be a placebo effect. Indeed, the Society for Interdisciplinary Placebo Studies defines placebo effects as changes specifically attributable to placebo mechanisms (e.g., neurobiological and psychological mechanisms of expectations). In other words, the placebo effect is a psychobiological response after a sham treatment (Hurst et al., 2017). Furthermore, in the last 2 decades, the placebo effect can also influence sports performance (Beedie et al., 2018). In this regard, Pollo et al. (2008) reported a significant increase in leg extension strength and a decrease in perceived fatigue when providing an ergogenic placebo. Similarly, Costa et al. (2019) observed that placebo provision was effective in improving bench press throw performance in Paralympic weightlifters. Moreover, performance in repeated 30-m sprints did not differ significantly between baseline and experimental trials when subjects believe in the effectiveness of a placebo treatment (Beedie et al., 2007). Although the precise mechanism of treatment is not fully understood, the individual’s belief in the efficacy of the treatment will most likely elicit positive changes and manifest the desired physiological and/or psychological benefit (Clark et al., 2000; Hurst et al., 2020). Therefore, it is crucial for those supporting athletes to reinforce the placebo effect of legitimate treatments by encouraging positive beliefs (Hurst et al., 2020). Similarly, as athletes and coaches commonly use CWI, there is a strong belief and/or expectation in the effectiveness of this treatment. Thus, the beneficial effect of CWI is associated with expectancy theory.

Despite the large number of studies concerning the effect of CWI on recovery, one of the main weaknesses of CWI researches to date is that very few studies have incorporated a Pla condition (Broatch et al., 2014; Gonzalez et al., 2014; Malta et al., 2018; Wilson et al., 2018; de Freitas et al., 2019; Wilson et al., 2019). These studies compared the effects of CWI and Pla condition on recovery following strenuous resistance exercise in resistance-trained males (Townsend et al., 2013; Gonzalez et al., 2014; Wilson et al., 2019), high-intensity interval exercise performed on an electromagnetic cycle ergometer (Broatch et al., 2014), a trail marathon in endurance-trained males (Wilson et al., 2018), and over a five consecutive day (i.e., microcycle) in volleyball players (de Freitas et al., 2019). Overall, CWI was not more effective than Pla condition in improving time course of recovery in these exercise modalities (Broatch et al., 2014; Wilson et al., 2018; de Freitas et al., 2019; Wilson et al., 2019). Indeed, these studies found no significant difference between CWI and Pla condition regarding indices of physical performance, muscle damage and inflammation, with the exception of CRP at 24 and 48 h after exercise (Wilson et al., 2018) and muscle edema on the successive day of volleyball training (de Freitas et al., 2019). However, despite the increased use of CWI in soccer and its popularity as the best recovery strategy, to our knowledge, no study to date has examined the magnitude of the placebo effect in improving recovery by CWI following a protocol designed to replicate the demands associated with intermittent activity in soccer. As it is well known that muscle damage mechanisms differ according to the nature of the exercise stress (Armstrong et al., 1991), and that soccer match is likely to result in higher metabolic stress and muscle damage responses compared to other team sports (Souglis et al., 2015), it would therefore be wise to examine the effect of CWI compared to a placebo condition in these particular conditions.

In this context, our study aimed to evaluate the effectiveness of CWI on recovery after LIST « as an intermittent running task that simulates the activity pattern of soccer » by investigating whether the placebo effect was implicated in this efficiency. Therefore, the main purpose of the present study was to directly compare the effects of CWI vs. Pla on recovery processes following LIST in semi-professional soccer players. Since CWI has been shown to improve recovery after LIST (Bailey et al., 2007; Bouzid et al., 2018) and there is common agreement among soccer players regarding its effectiveness (Hohenauer et al., 2015), we hypothesized that CWI would accelerate the recovery process after LIST, but that this effectiveness would be explained by the placebo effect.

## 2 Materials and methods

### 2.1 Participants

Twelve semi professional soccer players (age: 21.1 ± 2.2 years; body mass: 72.4 ± 5.9 kg; height: 174.9 ± 4.6 cm; $\dot{V}$ O_2max_: 56.1 ± 2.3 mL/min/kg) from a Tunisian senior squad, volunteered to take part in this study. They have played soccer at a rate of six training sessions including one official match per week for at least seven seasons and have used the CWI at least once a week during the season. We excluded any participants presenting allergy to cryotherapy, recent history of musculoskeletal injury or rehabilitation during the experimental period.

The study was conducted according to the Declaration of Helsinki and was approved by the local clinical Research Ethics Committee (CPP N: 0227/20). All included participants were fully informed about the objectives, risks and benefits associated to this study and provided written informed consent before testing. Finally, subjects were instructed to abstain from caffeine, alcohol consumption and intense exercise for 48 h prior to the first day of testing and lasting until the completion of the experimental protocol.

### 2.2 Experimental design

**FIGURE 1:**
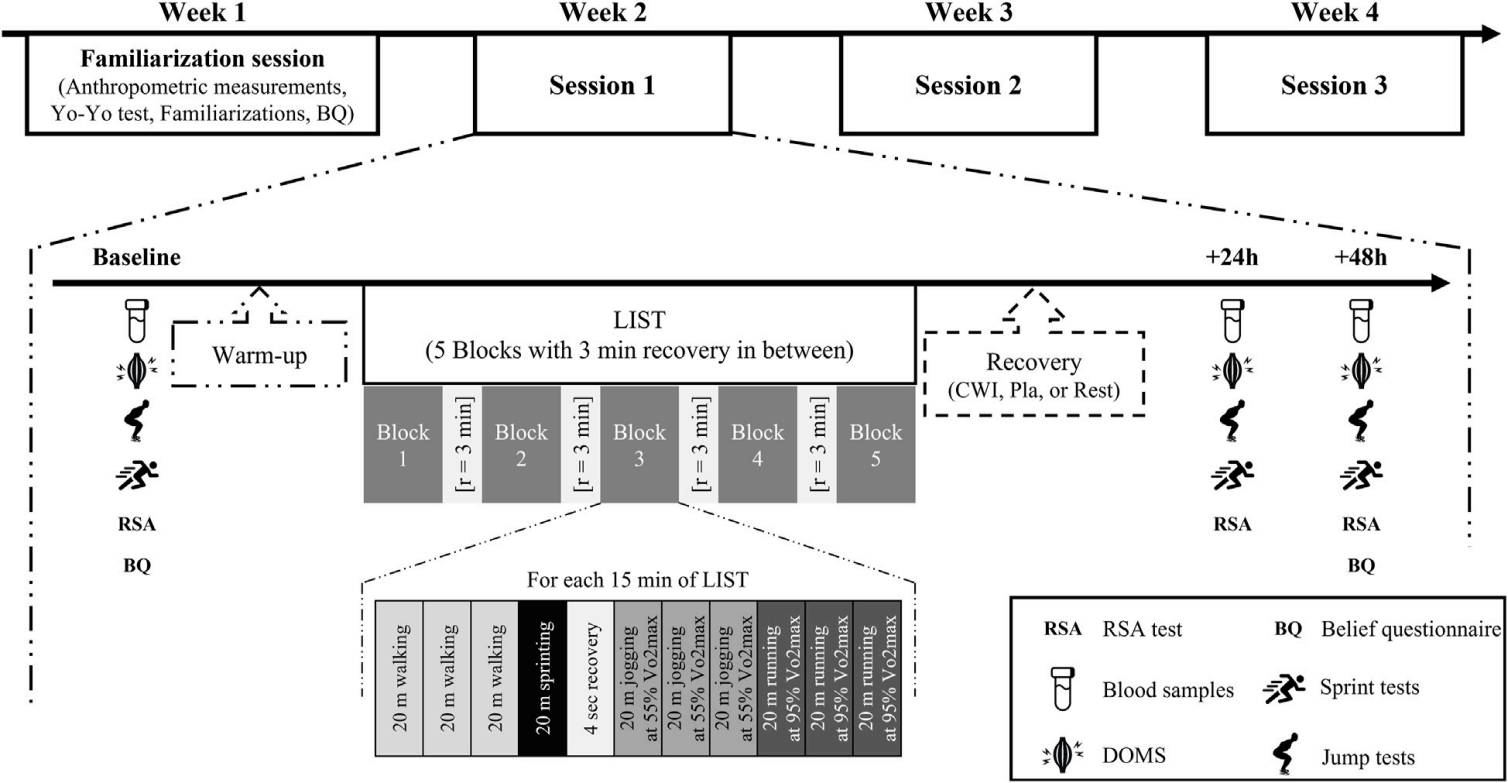
Experimental protocol.

Each participant performed four field sessions over a 4 week period (Figure 1). During the first week, participants anthropometric measurements were taken and they underwent the Yo-Yo intermittent recovery test level 1 (Yo-Yo IRT level 1) (Bangsbo et al., 2008). Prior to the familiarization session, an information sheet devoted to explain the efficiency of each recovery modality (CWI, Pla and Rest) was given to all participants.

#### 2.2.1 CWI

participants were fully informed that CWI is one of the most popular strategies used by soccer players, and might have a beneficial effect on recovery kinetics after LIST.

#### 2.2.2 Pla

participants were informed that the « recovery Pla beverage », has the same levels of effectiveness as CWI in promoting recovery and those they should expect the same listed beneficial outcomes.

#### 2.2.3 Rest

the participants were informed that Rest is not the best recovery strategy, especially that soccer players need to accelerate recovery process after LIST since it is considered to induce muscle damage.

Subsequently, they were familiarized with all the testing procedures at least 96 h before the baseline session. The study followed a randomized, counter-balanced design, in which each participant performed the three recovery conditions over three separate weeks. On the first day of each week, participants carried out a standardized warm-up of 9 min submaximal run at 8 km/h, 7 min of soccer-specific warm-up (changes of direction, sprints, and jumps) and dynamic stretching. Then, they performed the LIST followed by one of the recovery conditions (CWI, Pla and Rest). All variables were measured before (baseline) and 24 and 48 h after LIST.

### 2.3 Loughborough intermittent shuttle test (LIST)

The LIST is a field test designed to replicate the demands associated with intermittent activity such as soccer. Nicholas et al. (2000) have detailed all the procedures to be followed to perform LIST.

In an artificial surface soccer pitch, participants were required to run between two lines at 20 m apart with various speed dictated. The test consists of five sets of 15 min separated by 3 min of passive recovery; each 15-min interval includes the following exercise pattern: 3 × 20 m at walking pace, 1 × 20 m at maximal running speed, 4 s recovery, 3 × 20 m at a running speed corresponding to 55% of individual velocity at maximal oxygen uptake (v $\dot{V}$ O_2max_), and finally 3 × 20 m at a running speed corresponding to 95% of individual v $\dot{V}$ O_2max_.

The speeds of each player referred to the maximum running speed achieved in the Yo-Yo IRT level (v $\dot{V}$ O_2max_: 17.1 ± 0.8 km h^-1^) that was used to determine athlete’s ability to perform intense intermittent exercise performed during familiarization (Bangsbo et al., 2008).

### 2.4 Recovery interventions

All recovery modalities were performed into the changing rooms next to the field under standardized conditions (temperature: 23°–25°C; relative humidity: 40%–60%). Subjects sat comfortably and remained quiet and silent. In all conditions, participants drank the same amount of fruit-flavoured drink in non-transparent bottles labeled differently mentions (“fruit-flavored drink” for CWI and Rest conditions and “tart montmorency cherry juice” for the Pla condition). All modalities were conducted 5–10 min after LIST.

#### 2.4.1 Cold water immersion (CWI)

Participants wore only shorts and assumed a sitting position in an inflatable pool (legs straight and fully relaxed), up to the level of the sternum continuously for 15 min in cold water at 11.3°C ± 0.2°C, with a hydrostatic pressure of approximately ∼40 mmHg. The amount of pressure acting on a body is equal to (hydrostatic pressure = ambient pressure [standard sea level ∼1,013 hPa] + (gravity [9.81 m/s^2^] × water density [1,000 kg/m^3^] × immersion depth) (Wilcock et al., 2006). The water temperature was monitored minute by minute with a digital thermometer (212–130, RS Products, Texas, United States) and target temperatures (∼11°C) were achieved by adding ice to the cold water. The immersion duration and water temperature used in the present study were based on those proposed in a recent literature review on the effects of different CWI durations and temperatures (Machado et al., 2016). Indeed, these authors showed that protocols with temperatures between 11°C and 15°C for 10–15 min had a positive effect compared to “severe cold” immersion protocols with a temperature between 5°C and 10°C with an immersion time of less than 10 min.

Finally, participants were instructed to make circular movements with their legs every 2 min to prevent the formation of a warmer boundary layer surrounding the skin (Vieira et al., 2016).

#### 2.4.2 Placebo (Pla)

Participants consumed 0.4 L of a fruit-flavoured drink that contained no antioxidants or phytonutrients. They were asked to sit for 15 min and were asked to drink one-third of it every 5 min. They were told that it was a tart cherry juice supplement. Indeed, the phytochemicals in tart montmorency cherry juice have previously been shown to reduce inflammation and improve muscle recovery after a marathon (Bell et al., 2014).

#### 2.4.3 Rest recovery (Rest)

Participants were resting on a comfortable mat in the same position as in the CWI and Pla conditions for 15 min, with minimal movement and at the same room temperature.

### 2.5 Measurements

#### 2.5.1 Belief questionnaire (BQ)

Participants were asked to rate the effectiveness of the recovery modality on a five-point Likert scale ranging from 0 (indicating « not effective at all ») to (5 indicating « extremely effective »). Belief in the effectiveness of the recovery modality was evaluated before the start of familiarization (BQbf), just before participation in the experiment for each condition (session 1, 2, and 3) (BQpre), and at the end of each experimental condition in the protocol (BQpost).

#### 2.5.2 Biochemical markers

During soccer match, the large number of repeated maximal intermittent muscle actions cause a high metabolic stress (urea, ammonia and cortisol) and increased the muscle damage indices [creatine kinase (CK) and lactate dehydrogenase (LDH)], as well as inflammatory cytokines [tumor necrosis factor-a (TNFα) and interleukin-6] and C-reactive protein (CRP) (Souglis et al., 2015). Similarly, LIST induced a significant increase in CK activity and CRP (Magalhães et al., 2010; Leeder et al., 2015). In order to assess muscle damage and inflammation in the present study, blood samples (10 mL) were collected for each participant from the antecubital vein into an EDTA tube (Greiner Bio-One, Germany). Whole blood samples were centrifuged at 1,000–2,400 g for 15 min at 4°C, and then processed for plasma and stored at −80°C until analysis. Plasma CK and uric acid (UA) concentrations were analyzed spectrophotometrically using commercial test kits (A11A01632, Horiba-ABX, Montpellier, France; myoglobin bio Merieux 30446 and Roche Diagnostics, Carnaxide, Portugal, respectively). Serum CRP was measured using an enzyme-linked immune sorbent assay system (ELISA-PENTRA 400, Horiba ABX, Montpellier, France) and according to the manufacturers’ instructions. The intra-assay coefficient of variation for these parameters was 1.12%, 1.22%, and 2.16%, respectively. All biochemical tests were carried out in an accredited hospital laboratory with strict control of the corresponding techniques.

#### 2.5.3 Muscle soreness (DOMS)

Delayed onset muscle soreness (DOMS) was determined using a 20 cm visual analogue scale, with 0 cm corresponding to “no pain” and 20 cm corresponding to “extremely painful” as previously described (Leeder et al., 2014). Participants were asked to place their hands on their hips and performed a 90° knee flexion squat for 3 s before assessing their pain level.

#### 2.5.4 Jumping performance (SJ and CMJ)

Participants performed three trials for each jump form with 1 min of rest between trials. The best jump height from the three trials was recorded and used for data analysis. For both jumping techniques, the feet were shoulder width apart and the hands were placed on the hips. All jumps were performed in an indoor hall with jumping shoes worn using an Optojump system (Globus; Microgate, SARL, Italy). Verbal encouragement was given during trials.

For the squat jump (SJ), participants performed a maximal vertical jump with their hands on their waist, from a static position with a 90° knee flexion angle was held for 3 s before each jump attempt. No countermovement or arm swing was allowed during the jump.

For the countermovement jump (CMJ), participants performed a maximal vertical jump from an upright position and then performed a rapid downward movement by bending the knees and hips, immediately followed by a rapid leg extension resulting in a maximal vertical jump.

#### 2.5.5 Sprint tests (10 and 20 mS)

The 10 and 20 mS tests were performed 2 min after the jump test session on a soccer pitch (artificial grass) wearing soccer shoes. For all sprint tests, participants started from a standing position, with the toe of the foot preferred forward 0.2 m behind the starting line, and were instructed to perform all the sprints at maximal intensity. Three trials for each distance were performed with 1 min rest period between repetitions and the best performance from three trials was recorded for data analysis. Sprint times were recorded using a photocell system (Brower Timing Systems, Salt Lake City, Utah, United States). The cells were placed at 0.75 m above the ground and 2 m before the start and finish line.

#### 2.5.6 Repeated sprint ability (RSA)

The RSA test consisted of 12 × 20 m sprints interspersed with a 20 s passive recovery (Newman et al., 2004). For the first sprint, the players started freely. For the rest of the sprints, there was a 5-s count down at the end of the passive recovery. For each sprint, subjects started sprinting 0.2 m behind the starting line. The RSA performance was calculated by dividing the total sprint time by the number of sprints. The timing system and procedures were the same as for the sprint tests.

### 2.6 Statistical analysis

Data were presented as mean and standard deviation (SD). Statistical analysis was performed using STATISTICA (version 10.0, StatSoft, Inc., Tulsa, OK, United States) and data visualization was performed with GraphPad Prism (version 8.0.2 for Windows, GraphPad Software, Inc., San Diego, California, United States). Normality of the dependent variables was checked and confirmed using the Shapiro Wilk test. Two-way repeated measures analyses of variance (ANOVA) for BQ [Condition (CWI, Pla, and Rest) × Time (BQbf, BQpre and BQpost)] and for the other dependent variables [Condition (CWI, Pla, and Rest) × Time (Baseline, 24 h, and 48 h)] were used to determine differences between experimental conditions. Effect size data (η_p_2) was calculated to determine the magnitude of changes using the following criteria: 0.01, 0.06, and 0.14 represent small, moderate and large effect sizes, respectively (Cohen, 1988). When a significant difference was found, Fisher’s LSD *post hoc* test was used. Significance was accepted for all analyses, *a priori*, at the *p* < 0.05 level. For reliability of physical tests, the intraclass correlation coefficient (ICC) was calculated. ICC values over 0.75 were considered as excellent reproducibility, ICC values between 0.4 and 0.75 were considered as good reproducibility, and ICC values less than 0.4 was considered as poor reproducibility (Cohen, 2013).

## 3 Results

### 3.1 Belief questionnaire (BQ)

**FIGURE 2:**
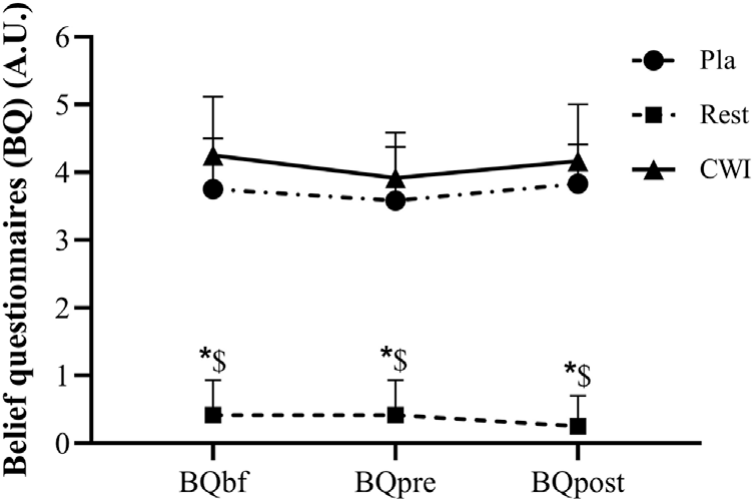
Belief questionnaire (BQ) score recorded before the start of familiarization (BQbf), before (BQpre) and post (BQpost) the experiment for each condition during placebo (Pla), rest recovery (Rest) and cold water immersion (CWI) conditions. *: significant difference in comparison to Pla (*p* < 0.05). $: significant difference in comparison to CWI (*p* < 0.05).

There was neither condition × time interaction (F_2,22_ = 1.77, *p* = 0.19, η_p_2 = 0.14) nor a main effect of time (F_1,11_ = 3.14, *p* = 0.10, η_p_2 = 0.22). Nevertheless, a main effect of condition was found (F_2,22_ = 202.54, *p* < 0.001, η_p_2 = 0.95), with lower values in Rest condition at baseline and 48 h after LIST compared to Pla (*p* = 0.001; 95%CI = 2.60, 3.73 and *p* = 0.001; 95%CI = 4.14, 5.01, respectively) and to CWI conditions (*p* < 0.001; 95%CI = 3.00, 3.99 and *p* < 0.001; 95%CI = 3.34, 4.47, respectively). However, no significant difference was found between CWI and Pla at baseline (*p* = 0.078) and 48 h after LIST (*p* = 0.078) (Figure 2).

### 3.2 Biochemical markers

#### 3.2.1 CK

**FIGURE 3:**
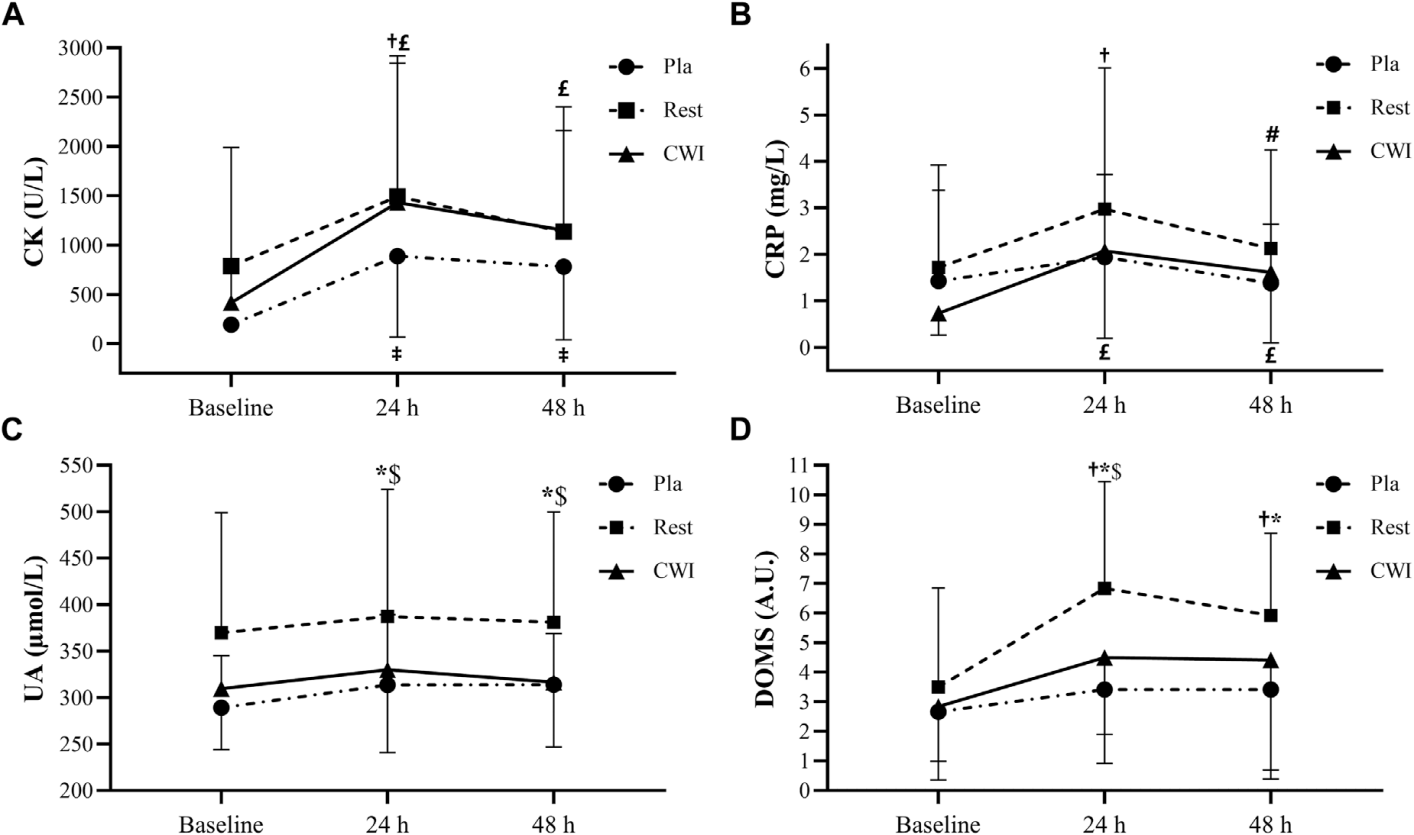
Plasma creatine kinase (CK) **(A)**, C-reactive protein (CRP) **(B)**, uric acid (UA) **(C)**, and delayed onset muscle soreness (DOMS) **(D)** values recorded at baseline, at 24 and 48 h following LIST during placebo (Pla), rest recovery (Rest) and cold water immersion (CWI) conditions.‡: significant difference compared with baseline values for Pla (*p* < 0.05). †: significant difference compared with baseline values for Rest (*p* < 0.05). #: significant difference compared with 24 h values for Rest (*p* < 0.05). £: significant difference compared with baseline values for CWI (*p* < 0.05). *: significant difference between Pla and Rest values (*p* < 0.05). $: significant difference between CWI and Rest values (*p* < 0.05).

There was neither condition × time interaction (F_4,44_ = 0.92, *p* = 0.46, η_p_2 = 0.08), nor a main effect of condition (F_2,22_ = 1.14, *p* = 0.33, η_p_2 = 0.09). Nevertheless, a main effect of time was found (F_2,22_ = 14.21, *p* < 0.001, η_p_2 = 0.56). CK increased significantly from baseline to 24 h after LIST in Rest condition (*p* = 0.001; 73.99%; 95%CI = −381.73, 1794.33), but not from baseline to 48 h (*p* = 0.09). Likewise, CK increased significantly from baseline to 24 and 48 h after the LIST for Pla (*p* = 0.005; 358.81%; 95%CI = 201.77, 1,190.23 and *p* = 0.019; 302.62%; 95%CI = 141.13, 1,032.87, respectively) and for CWI (*p* < 0.001; 243.14%; 95%CI = 142.12, 1883.67 and *p* < 0.001; 176.58%; 95%CI = −40.01, 1,511.61, respectively) (Figure 3).

#### 3.2.2 CRP

There was neither condition × time interaction (F_4,44_ = 1.27, *p* = 0.3, η_p_2 = 0.10), nor a main effect of condition (F_2,22_ = 0.80, *p* = 0.46, η_p_2 = 0.068). However, a main effect of time was found (F_2,22_ = 6.02, *p* = 0.008, η_p_^2^ = 0.356). Indeed, CRP increased significantly from baseline to 24 h and decreased significantly from 24 to 48 h after LIST for Rest condition (*p* = 0.002; 73.17%; 95%CI = −0.46, 3.00 and *p* = 0.024; −28.44%; 95%CI = −1.04, 2.74 respectively). Furthermore, CRP increased significantly from baseline to 24 h (*p* = 0.001; 182.95%; 95%CI = 0.18, 2.49) and 48 h (*p* = 0.01; 120.45%; 95%CI = −0.07, 1.83) after the LIST in the CWI condition. Finally, there was no significant difference between the three time-points in Pla condition (*p* > 0.05).

#### 3.2.3 UA

There was neither condition × time interaction (F_4,44_ = 0.73, *p* = 0.58, η_p_2 = 0.06), nor a main effect of time (F_2,22_ = 2.77, *p* = 0.08, η_p_^2^ = 0.35). However, a main effect of condition was found (F_2,22_ = 5.89, *p* = 0.009, η_p_^2^ = 0.35), with higher values in Rest compared with Pla and CWI conditions at 24 h (*p* < 0.001; 95%CI = −19.08, 166.44 and *p* < 0.001; 95%CI = −32.04, 146.32, respectively) and 48 h (*p* < 0.001; 95%CI = −14.60, 148.76 and *p* < 0.001; 95%CI = −13.24, 142.16, respectively) after LIST.

### 3.3 Delayed onset muscle soreness (DOMS)

There was no significant condition × time interaction (F_4,44_ = 0.1, *p* = 0.42, η_p_2 = 0.08). However, a main effect of condition and time was found (F_2,22_ = 4.47, *p* = 0.023, η_p_^2^ = 0.29; F_2,22_ = 7.50, *p* = 0.003, η_p_^2^ = 0.41, respectively). The *post hoc* test showed that DOMS scores were higher in Rest condition compared with Pla condition at 24 h (*p* = 0.001; 95%CI = 0.79; 6.04) and 48 h (*p* = 0.001; 95%CI = 0.04, 4.95) after LIST but only at 24 h after LIST compared with CWI condition (*p* = 0.017; 95%CI = −0.33, 4.99). Moreover, DOMS scores increased significantly from baseline to 24 h (*p* = 0.001; 95.24%; 95%CI = 0.38, 6.27) and 48 h (*p* = 0.014; 69.05%; 95%CI = −0.18, 5.00) after LIST, only for Rest condition (Figure 3).

### 3.4 Jumping performance

#### 3.4.1 SJ

Statistical analysis showed an interaction between condition and time (F_4,44_ = 2.71, *p* = 0.042, η_p_^2^ = 0.2) and a main effect of time (F_2,22_ = 6.27, *p* = 0.007, η_p_^2^ = 0.36). Indeed, SJ performance decreased significantly from baseline to 24 h (*p* = 0.001; −7.24%; 95%CI = −0.29, 5.49) and 48 h (*p* < 0.001; −9.19%; 95%CI = 0.72, 5.81) after LIST at Rest condition. However, there was no significant difference between the three time-points in the Pla and CWI conditions (Table 1).

**TABLE 1:** Squat jump (SJ), countermovement jump (CMJ), 10-m sprint (10 mS), 20-m sprint (20 mS) and repeated sprint ability (RSA) recorded at baseline, 24 and 48 h following LIST for the three recovery conditions [placebo (Pla), rest recovery (Rest) and cold water immersion (CWI)].

Variables	Conditions	Time		
		Baseline	24h	**48h**
SJ (cm)	**Pla**	34.03 ± 3.96	32.83 ± 3.34	32.9 ± 3.21
	**Rest**	35.8 ± 2.81	33.21 ± 3.25‡	32.53 ± 3.2‡
	**CWI**	34.03 ± 4.68	34.28 ± 4.26	33.58 ± 2.29
CMJ (cm)	**Pla**	36.26 ± 4.02	35.32 ± 3.24	34.65 ± 3.56‡
	**Rest**	37.31 ± 3.26	35.28 ± 2.59‡	35.18 ± 3.66‡
	**CWI**	35.95 ± 4.58	35.99 ± 4.33	36.24 ± 3.5
10 mS (s)	**Pla**	1.98 ± 0.14	1.93 ± 0.08	1.91 ± 0.11‡
	**Rest**	1.93 ± 0.07	1.97 ± 0.06*	1.95 ± 0.09
	**CWI**	1.91 ± 0.1	1.98 ± 0.09‡*	1.91 ± 0.09†
20 mS (s)	**Pla**	3.41 ± 0.21	3.34 ± 0.14	3.38 ± 0.17
	**Rest**	3.26 ± 0.12	3.33 ± 0.35	3.37 ± 0.17
	**CWI**	3.35 ± 0.18	3.37 ± 0.2	3.32 ± 0.15
RSA (s)	**Pla**	3.32 ± 0.11	3.31 ± 0.07	3.37 ± 0.17‡†
	**Rest**	3.31 ± 0.06	3.42 ± 0.09‡*	3.36 ± 0.12†
	**CWI**	3.36 ± 0.16	3.38 ± 0.14*	3.33 ± 0.1

‡: significant difference compared with baseline values (*p* < 0.05). †: significant difference compared with 24 h values (*p* < 0.05). *: significant difference compared with Pla condition (*p* < 0.05).

#### 3.4.2 CMJ

For CMJ performance, there was neither condition × time interaction (F_4,44_ = 2.24, *p* = 0.08, η_p_2 = 0.17), nor a main effect condition (F_2,22_ = 0.96, *p* = 0.39, η_p_^2^ = 0.08). However, a main effect of time was found (F_2,22_ = 6.19, *p* = 0.007, η_p_^2^ = 0.36). The *post hoc* test revealed that CMJ performance decreased significantly from baseline to 24 h (*p* = 0.003; −5.45%; 95%CI = −0.45, 4.51) and 48 h (*p* = 0.002; −5.70%; 95%CI = −0.81, 5.05) after LIST for Rest condition, but only from baseline to 48 h after LIST for Pla condition (*p* = 0.019; −4.43%; 95%CI = −1.60, 4.80). Finally, no significant difference was found between the three time-points in CWI condition (*p* > 0.05).

### 3.5 Sprint performance

There was an interaction between condition and time for the 10 mS performance (F_4,44_ = 3.67, *p* = 0.011, η_p_^2^ = 0.287), but not for the 20 mS performance (F_4,44_ = 1.1, *p* = 0.36, η_p_^2^ = 0.09).

In the Pla condition, 10 mS performance increased significantly from the baseline to 48 h after LIST (*p* = 0.016; −3.16%; 95%CI = −0.03, 0.15), with no significant difference between baseline and 24 h. For CWI condition, 10 mS performance decreased significantly from baseline to 24 h after LIST (*p* = 0.003; +4.11%; 95%CI = 0.03, 0.14), then increased significantly from 24 h to 48 h (*p* = 0.006; −3.65%; 95%CI = 0.00, 0.13). However, no significant difference between the three time-points was observed in the Rest condition. Moreover, 10 mS performance was significantly better in Pla condition than in Rest (*p* < 0.001; 95%CI = −0.01, 0.07) and in CWI (*p* = 0.021; 95%CI = −0.04, 0.06) conditions only at 24 h after LIST (Table 1).

### 3.6 Repeated sprint ability (RSA)

Statistical analysis showed an interaction between condition and time for the RSA performance (F_4,44_ = 4.45, *p* = 0.004, η_p_^2^ = 0.287). Indeed, RSA performance decreased significantly from the baseline to 48 h (*p* = 0.043; +1.79%; 95%CI = −0.06, 0.18) and from 24 to 48 h (*p* = 0.03; +1.90%; 95%CI = −0.05, 0.17) after LIST in Pla condition. In the Rest condition, RSA performance decreased significantly from the baseline to 24 h after LIST (*p* < 0.001; +3.35%; 95%CI = 0.04, 0.17), then increased significantly from 24 to 48 h (*p* = 0.021; −1.98%; 95%CI = −0.02, 0.16). However, no significant difference was found between the three time-points in CWI condition (*p* > 0.05). Furthermore, RSA performance was significantly better in Pla condition than in Rest (*p* < 0.001; 95%CI = 0.03, 0.18) and in CWI (*p* = 0.021; 95% CI = −0.02, 0.16) conditions only at 24 h after LIST (Table 1).

### 3.7 Reliability

The reliability of the physical tests was evaluated using the baseline test results for each condition. The ICC showed excellent reliability for RSA (ICC (95% CI) = 0.78 (0.43, 0.93)), SJ (ICC (95% CI) = 0.89 (0.73, 0.96)), and CMJ (ICC (95% CI) = 0.93 (0.82, 0.97)) and poor reliability for 20 mS (ICC (95% CI) = 0.21 (−1.07, 0.75)) and 10 mS (ICC (95% CI) = 0.01 (−1.60, 0.69)).

## 4 Discussion

To our knowledge, this is the first study to compare the effect of CWI and placebo intervention on recovery in semi-professional soccer players following LIST. As expected, the LIST protocol led to reductions in physical performance, increases in blood markers of muscle damage and inflammation and muscle soreness. These changes in physical, biochemical and psychological parameters explain the emergence of significant fatigue after LIST (Magalhães et al., 2010). The main result of the present study indicates that CWI and placebo were more effective than the Rest condition in recovery. Overall, the comparisons between CWI and Pla did not show remarkable differences in favour of CWI.

The use of a BQ in the present study allowed us to control whether the used Pla condition respected its expected effect. The BQ score was significantly lower in the Rest condition than in the Pla and CWI conditions at all times points. Moreover, no significant difference on the BQ score was found between CWI and Pla at all measurement times. Therefore, the information sheet explaining the effectiveness of each recovery modality has played its role effectively. Indeed, it misleads participants into believing that they received a beneficial treatment. The use of a Pla condition in the present study plays a major role in the success of the interventions, as it may negate some of the positive expectancy effects attributable to Pla effect (McClung and Collins, 2007; Broatch et al., 2014).

### 4.1 Recovery of physical performance

Jumping abilities are frequently used to monitor fatigue status and recovery kinetics in team sports, following EIMD, training and competition (Ingram et al., 2009). Our result showed that SJ performance decreased at 24 and 48 h after LIST compared with baseline value only for the Rest condition. Furthermore, there were no significant differences between CWI and Pla conditions at all measurement times. On the other hand, CMJ recorded the same result, with the exception for the Pla condition, which showed a significant decrease in jump height at 48 h compared to the baseline value. These results indicate that both CWI and Pla interventions accelerated recovery after LIST compared to the rest condition, thus making it possible to reproduce similar jumping performances (with the exception of CMJ performance at 48 h for the Pla condition, which will be discussed at the end). These findings are in accordance with the study of Bouzid et al. (2018) who showed that CWI (10 min at 10°C) facilitated SJ and CMJ recovery compared to TWI (10 min at 28°C) at 24, 48, and 72 h after LIST in professional soccer players. It has been already suggested that faster recovery of physical performance after CWI could be, at last partly, related to a lower level of muscle damage (Ascensão et al., 2011; Ihsan et al., 2016). However, our results regarding muscle damage markers do not support a reduction in muscle damage level after CWI. This finding confirms the conclusions of Bouzid et al. (2018) suggesting that these beneficial effects on physical performance recovery would not be related to CWI-induced lower muscle damage. More interestingly, looking at the comparison between CWI and Pla condition, our results indicate that this faster physical performance recovery after CWI is placebo effect related. In the same way, CWI (10 min at 10°C) had a trivial or reduced impact on CMJ recovery compared to Pla (a cornstarch pill) after acute resistance exercise (Wilson et al., 2019). Furthermore, CWI had no effect on recovery of jumping performance 24 h or more after LIST (Bailey et al., 2007; Leeder et al., 2015), a soccer match (Ascensão et al., 2011), and consecutive days of intermittent sprint exercise (King and Duffield, 2009). Otherwise, the decrease in CMJ performance only 48 h after LIST for the Pla condition may be explained by the importance of the stretch-shortening cycle mechanism in the CMJ (Byrne and Eston, 2002). Indeed, the SJ test is more sensitive than the CMJ and could be a valuable assessment to examine the rate of strength development without the stretch-shortening cycle (Byrne and Eston, 2002; Riggs and Sheppard, 2009).

Our results regarding sprint performance are difficult to interpret. Overall, our results suggest that 24–48 h would be sufficient for complete recovery of sprint performance after LIST regardless of the recovery condition. These results are in agreement with those of Ascensão et al. (2011) who reported that CWI has no effect on 20 mS compared to TWI (10 min at 35°C) throughout the recovery period after a soccer match. Furthermore, in professional soccer player, Bouzid et al. (2018) reported that 20 mS decreased at 24 h and returned to baseline levels at 48 h after LIST in both CWI and TWI conditions. Since short-distance sprinting ability is an important determinant for winning actions in a soccer match (Nédélec et al., 2012) and is the most frequent action before goals (Faude et al., 2012), the effects of CWI on sprinting ability needs to be fully elucidated. On the other hand, our results show that CWI and Pla conditions accelerated the RSA performance recovery at 24 h compared to the Rest condition after LIST, and that this beneficial effect could be placebo effect-related. These findings confirm those of Elias et al. (2012) who found that CWI (14 min at 12°C) facilitated restoration of RSA performance (6 × 20 m) compared with passive recovery only at 24 h after training session in Australian footballers. Nevertheless, Rowsell et al. (2009) showed no significant difference in the CWI condition (5 × 60 s at 10°C with 60 s between immersions) compared to the TWI condition (5 × 60 s at 34°C with 60 s between immersions) in RSA (12 × 20 m) performance recovery from a 4 days football tournament. Lack of or moderate benefits of CWI on RSA and sprint performance compared to jumping performance are thought to be related to the time course of recovery of different aspects of performance (Delextrat et al., 2013). Indeed, in the absence of a recovery intervention, full recovery of performance after a match was fastest for sprint performance (5 h), followed by strength (27–51 h) and finally jumping performance (over 69 h) in female soccer players (Andersson et al., 2008). Therefore, Delextrat et al. (2013) concluded that recovery interventions are more likely to affect jumping performance compared to sprinting performance, as a longer time would be required for recovery of this aspect of performance. Again, given the RSA performance importance in soccer specifically and in team sports in general (Bishop, 2010), the effect of CWI and these mechanisms still needs further investigation.

### 4.2 Recovery of biochemical markers

Regarding biochemical markers, CK was not significantly different between the three recovery conditions at all measurement times. On the other hand, CK was significantly higher at 24 h than at baseline in all conditions and remained significantly higher at 48 h than at baseline in both CWI and Pla conditions. These results clearly demonstrate that LIST caused muscle damage and that the CWI and Pla conditions were not able to decrease cell damage at 48 h after LIST. Our results are in agreement with some studies that reported a significant increase in CK at 24 and 48 h compared to baseline after CWI (10 min at 10°C) after LIST (Bouchiba et al., 2022) and a soccer match (Ascensão et al., 2011). However, with the same CWI immersion protocol (10 min at 10°C), it was observed that CWI attenuated the increase in CK activity at 48 h post-LIST compared to TWI in professional soccer players (Bouzid et al., 2018). Since we used the same EIMD (LIST) in a similar population with this latest study, these conflicting results can only be explained by the different treatment protocols such as water temperature and immersion duration (15 min at 11°C vs. 10 min at 10°C). Thus, it seems that the choice of temperature and immersion duration during CWI is very sensitive (Sanchez-Urena et al., 2017; Anderson et al., 2018). However, plasma UA another indicator used in our study to quantify muscle membrane disruption and purine metabolism, has been reported to increases after intense exercise (Palacios et al., 2015). The UA concentration did not vary significantly between the three measurements (baseline, 24 and 48 h) for all conditions. These results suggest that LIST was not intense enough to increase the UA concentration at 24 h or even 48 h post-LIST.

CRP activity plays an important role in the muscle recovery process and its concentration can give an indication of the inflammatory state (Pepys and Hirschfield, 2003). Our results indicate that CRP was not significantly different between the three conditions for all measurement times. Furthermore, CRP increased at 24 h for the Rest condition and at 24 and 48 h for CWI compared with baseline values, but no significant change was observed for the Pla condition. Thus, it seems that the CWI protocol in our study (15 min at 11°C) was not effective in reducing inflammation that could be explained by a cold related stress response (Machado et al., 2016). If our results confirm those of White et al. (2014) who found that CWI (10 min at 10°C) did not reduce plasma markers of inflammation after high-intensity sprint exercise, they contrast with the observations of Wilson et al. (2018) who reported that CWI (10 min at 8°C) had a positive effect on CRP activity compared to the Pla group (2 × 30 mL per day of a fruit flavoured drink for 8 days) at 24 and 48 h after completion of a trail marathon. These controversial results regarding the effects of CWI on CRP could be due to the EIMD used (LIST vs. marathon). Indeed, these two exercises are different in terms of metabolic factors such as exercise intensity and duration, glycogen depletion, dehydration, environmental conditions and external factors. High intensity intermittent exercise elicited a different total energy expenditure during exercise compared to endurance exercise (Cabral-Santos et al., 2015). Of particular interest is that the factor that may have the greatest impact on exercise-induced inflammatory responses is workload, which is the synergy between duration and intensity (Pedersen, 2009). Nevertheless, in the present study, CRP increased significantly from baseline to 24 h after the LIST in Rest and CWI conditions (73% and 183%, respectively) and CK increased significantly from baseline to 24 h after LIST in Rest, Pla and CWI conditions (74%, 359%, and 243%, respectively). Although these increases are significant and demonstrate the presence of muscle damage and inflammation after LIST, they are lower than those observed by Souglis et al. (2015). Indeed, these authors observed that CRP and CK peaked in the following morning after the match (13 h post-match), with the increase of 290% and 370% from baseline, respectively. It thus seems that the moment of measurement is important to take into account in the interpretation of the results.

### 4.3 Recovery of muscle soreness

LIST induced a significant increase in the level of muscle soreness only in the Rest condition. Indeed, DOMS score increased significantly from baseline to 24 and 48 h after LIST and was significantly higher in Rest condition compared to CWI and Pla conditions at 24 h and only compared to Pla at 48 h. Moreover, no significant difference was observed between CWI and Pla conditions for all measurement times. Our results are consistent with the observations of Hohenauer et al. (2020) who found a lower soreness perception in the CWI condition (10 min at 10°C) compared to the Rest condition after 5 × 20 drop jumps in females. These results demonstrate that CWI reduces muscle soreness and facilitates recovery from intermittent exercise and may therefore be useful in the prevention of muscle injuries. Indeed, a high level of DOMS may lead to an increased risk of injury (Cheung et al., 2003). Since Pla has the same results as CWI regarding DOMS, this suggests that the beneficial effect of CWI on DOMS is purely psychological, and is therefore none other than the placebo effect. According to our outcomes, the Pla condition seems to have an “ergogenic effect”, which reinforces the importance of considering the placebo effect in sports performance. Therefore, it is possible that the expectation of having received an ergogenic effect produces less muscle pain. Indeed, the potential mechanism of the placebo effect falls into four categories of “changes”: pain reduction, belief-behaviour relationship, attentional changes and arousal changes (Beedie et al., 2006). The improvements seen in our study can be explained by the reduction in pain. Indeed, the results of Hurst et al. (2020) showed that if an athlete does not fully believe in the efficacy of a “real” treatment, that athlete may not fully benefit from it. These authors suggest that the staff around athletes should strive to maximize the placebo effect of a legitimate treatment by generating a positive belief in its efficacy.

The vast majority of studies compared the effectiveness of CWI to a control condition and did not take into account the expectation effect or treatment belief. This is presumably due to the difficulty to blind participants to their recovery intervention. In the present study, in order to assess the effectiveness of CWI compared to an effectively delivered placebo intervention, we used a fruit-flavoured drink and an information sheet to mislead participants into believing they had received a beneficial treatment. Indeed, this strategy has already demonstrated its effectiveness (Wilson et al., 2018). Moreover, the BQ score confirmed the effectiveness of our strategy. However, failing to blind the examiners to the Pla condition, we have taken every precaution to not influence participants.

This study has some limitations that need to be addressed. Firstly, due to the difficulty of including professional athletes and excluding some participants during the experiment due to injury, the sample size is small. Secondly, to the best of our knowledge, no previous study has used the protocol of 15 min of CWI at 11°C, muscle and skin temperatures, heart rate and heart rate variability that may provide useful information on the heat exchange afforded by each recovery modality were not evaluated. Finally, a larger number of measurement acquisition time points (immediately after recovery, 72 and 96 h after LIST) would have provided a more complete view of the time course of recovery.

## 5 Conclusion

Our results indicate that CWI accelerated jumping and RSA performance recovery after LIST and that these beneficial effects would not be related to CWI-induced lower muscle damage but was placebo effect related. Indeed, CWI was not able to decrease LIST-induced muscle damage and inflammation at 48 h after LIST. Moreover, our results demonstrate that CWI reduces muscle soreness and that the beneficial effect of CWI on DOMS was related to the placebo effect. Nevertheless, 24 h would be sufficient for sprint performance complete recovery after LIST regardless of the recovery condition. Overall, these data confirm that the benefits of CWI may be at least partly due to a placebo effect and that the effectiveness of CWI in recovery after an intermittent exercise such as LIST can be explained by the strong belief in soccer players that it will be beneficial. Furthermore, it would be valuable to use other exercises as well as other markers of recovery in order to better understand the influence of the placebo effect in improving recovery after CWI in future research.

## Data Availability

The original contributions presented in the study are included in the article/supplementary material, further inquiries can be directed to the corresponding author.

## Abbreviations

10 mS, 10-m sprint; 20 mS, 20-m sprint; BQ; belief questionnaire; CK, creatine kinase; CMJ, countermovement jump; CRP, C-reactive protein; CWI, cold water immersion; DOMS, delayed onset muscle soreness; LIST, Loughborough Intermittent Shuttle Test; MVC, maximal voluntary contraction; Pla, placebo; Rest, passive recovery; RSA, repeated sprint ability; SJ, squat jump; TWI, thermoneutral water immersion; UA, uric acid.
